# Robust Model-Free Adaptive Iterative Learning Control for Vibration Suppression Based on Evidential Reasoning

**DOI:** 10.3390/mi10030196

**Published:** 2019-03-19

**Authors:** Liang Bai, Yun-Wen Feng, Ning Li, Xiao-Feng Xue

**Affiliations:** 1 School of Aeronautics, Northwestern Polytechnical University, Western Youyi Street 127, Xi’an 710072, China; fengyunwen033616@163.com (Y.-W.F.); xuexiaofeng033616@163.com (X.-F.X.); 2 College of Sciences, Northeastern University, Shenyang 110819, China; lining80@163.com

**Keywords:** P-type IL, MFA control, SM control, evidence theory, active vibration control, piezoelectric smart structure

## Abstract

Through combining P-type iterative learning (IL) control, model-free adaptive (MFA) control and sliding mode (SM) control, a robust model-free adaptive iterative learning (MFA-IL) control approach is presented for the active vibration control of piezoelectric smart structures. Considering the uncertainty of the interaction among actuators in the learning control process, MFA control is adopted to adaptively adjust the learning gain of the P-type IL control in order to improve the convergence speed of feedback gain. In order to enhance the robustness of the system and achieve fast response for error tracking, the SM control is integrated with the MFA control to design the appropriate learning gain. Real-time feedback gains which are extracted from controllers construct the basic probability functions (BPFs). The evidence theory is adopted to the design and experimental investigations on a piezoelectric smart cantilever plate are performed to validate the proposed control algorithm. The results demonstrate that the robust MFA-IL control presents a faster learning speed, higher robustness and better control performance in vibration suppression when compared with the P-type IL control.

## 1. Introduction

As an intelligent control strategy, iterative learning (IL) control has a simple structure and doesn’t require accurate system modeling. According to past control experience, this method can improve the current control performance of the system by operating repetitively over a fixed time interval [1]. In 1978, IL control was first proposed by Uchiyama in Japanese [2], which did not receive much attention. After one critical report published by Arimoto in English [3], IL control made significant progress in both theories and applications [4,5]. Practically, IL control has been applied to a wide range of engineering applications, including flexible structures [6], nonholonomic mobile robots [7], flapping wing micro aerial vehicles [8] and the sheet metal forming process [9]. Considering the repeatability of the structural dynamic response in the vibration process, IL control is expected to provide a feasible solution for the control issue here. Several research groups have applied IL control methods to the active vibration control of piezoelectric smart structures. Zhu et al. [10] and Tavakolpour et al. [11] first applied P-type IL control for the attenuation of vibrations in piezoelectric smart structures for the design of the feedback gain, and the efficiency of P-type IL control was proven in their papers. In addition, Fadil et al. [12] proposed a new intelligent proportional-integral-derivative (PID) controller for vibration suppression by using P-type IL control and PID control, in which the P-type IL control was applied to tune the parameters of the PID controller. 

In the studies above, although P-type IL controllers can effectively attenuate structural vibrations at some excitation frequencies, the performances of the controllers for vibration suppression are still not obvious when the piezoelectric smart structure is excited by its first natural frequency. Besides, the control effectiveness of the actuators is obvious at the locations of the sensors, and they are not able to effectively compensate for unwanted vibrations at other locations [13]. Moreover, thousands of iterations in P-type IL control are needed for achieving satisfactory control precision, leading to the slow learning speed [11,14]. In addition, the learning process should be accomplished within a limited period, as overlearning may lead to system instability [11]. Therefore, the iterative number should be limited to a predefined value. Finally, the unreasonable selections of learning gains may directly lead to control spillover or system instability [15]. 

For accelerating the convergence rate and improving the stability of the learning algorithm, adaptive control is needed to modify the learning process of the P-type IL control. Adaptive control has been successfully incorporated into various learning algorithms for the adjustment of parameters of the learning process [16,17]. Effective adaptive control strategies are necessary for the ability to automatically tune parameters to the desired performance at each sampling period, such as an internal model control method with an adaptive algorithm, implemented to reduce fatigue loads and tower vibrations in wind turbines [18]. A real-time control implementation, based on an auto-tuning finite impulse response filter, was applied to active vibration isolation [19]. An online method that tunes the poles of the controller was proposed to adapt to the errors between a real object and its model [20]. An adaptive voltage and frequency control method was proposed for inverter-based distributed generations in a multi-microgrid structure [21]. A characteristic model-based nonlinear golden section adaptive control method was presented for vibration suppression in a flexible Cartesian smart material robot [22].

As mentioned above, most of the adaptive control methods are model-based, in which the dynamic model of system has been already known before the design of the controller. However, for complex practical systems, the mechanism models of the plants are often difficult to establish, and the parameters are also hard to identify, making the design and application of controllers unpractical. Controlling vibrations in plate and shell structures always brings a challenge because of the complexity and density of the vibration modes. The strategy of using piezoelectric actuator-sensor pairs with discrete locations, glued on both surfaces of the plate, realizes a low weight and effective control for structural vibration [23]. The plate-integrated piezoelectric actuator-sensor pairs thus become a multi-input-multi-output (MIMO) system. If an actuator fails to perform as expected, the performance of its neighboring actuators will be negatively affected. In this system, the interaction among all actuators exists in the whole process of active vibration control, and this kind of interaction is always uncertain. The uncertainty caused by this interaction greatly presents a great challenge when designing a controller, and the model-based adaptive controller cannot deal with these conditions. Data-driven control methods, which are designed by directly using input and output (I/O) data of the system, can serve as an efficient alternative. The control problems caused by time-varying parameters and uncertainties of the model are challenging for model-based control, but not with data-driven control approaches [24]. 

Model free adaptive (MFA) control, as an effective data-driven control method, is an attractive technique which has gained a large amount of interest in recent years. It is easily implemented, with small computational burden for its simple structure and strong robustness. Unlike the neural-network-based adaptive control methods and model-based methods, no additional signal testing or training processes are required during the design of data-driven control methods. Instead of identifying the model of the plant, the MFA method builds an equivalent linearization of the data at each operation by introducing a novel concept named the pseudo-partial derivative (PPD), and the time-varying PPD can be estimated by merely using the I/O measurements of the plant.

In this paper, in order to accelerate the convergence speed of the feedback gain, the learning gain of the P-type IL control is designed by the MFA control. The MFA control can realize adaptive control in parametric and structural manners. And it is also suitable for dealing with system uncertainties [25]. This advantage allows MFA control to be successfully employed in various engineering fields, such as use in the sensing and control of piezoelectrically actuated systems [26], blood pump control [27], multivariable industrial processes control [28], and robotic exoskeleton tracking control [29]. However, the convergence speed of the tracking error may be slow if only MFA control is used to adaptively adjust to the learning gain of the P-type IL control, and external noise in the system will increase the difficulty of vibration control. Various control strategies have been provided and continuously developed for the control of plants with unknown uncertainties and dynamic variations, such as sliding mode (SM) control [30], fuzzy logic control [31], neural networks [32], etc. For nonlinear, time-varying and uncertain systems, neural network approaches have an excellent approximation ability, and fuzzy logic control possesses remarkable robustness and adaptability, nevertheless, the tuning of numerous parameters and complex rules may decrease the efficiency and possibility of these methods [33]. Unlike neural networks and fuzzy logic control, SM control has a simple controller structure and can is easily implemented. Moreover, it has other attractive features, including good transient performance, robustness to parameter variations and insensitivity to disturbances [34]. With the aim of achieving a faster response and better robustness, the SM control was integrated with the MFA control to self-adjust the learning gain of the P-type control. Within the proposed method, the SM control is applied to estimate the parameters by tracking the time-varying PPD, such that the state variables can rapidly converge to the desired trajectory. Additionally, SM control can also be used to compensate for the impact of random disturbance, thereby enabling the system to enhance control effectiveness and maintain superior stability. In the P-type IL algorithm, two parts mainly affect the convergence speed of the feedback gain: The system output error and the learning gain. In the application of vibration suppression, the desired output signal is always zero. Therefore, the measured output signal is the main factor that decides the system output error. In this paper, multi-sensors are used to detect the structural deformation of a cantilever plate. Sensors at various locations generate different measured output signals, and the controllers connected to these sensors may have distinct learning speeds. It is unreasonable to define the same iterative number for all controllers as part of the stopping criteria. After obtaining multi-source information from the controlled plant, it is critical to discover the optimal method of fusing this information. In this paper, in order to solve the multicriteria and multiobjective problems in practical applications, evidence theory was adopted to design the stopping criteria. The evidence theory does not require prior knowledge and has outstanding performance for handling uncertain or inexact information, which makes it an indispensable tool for state diagnosis and defect inspection [35,36]. Applying the combination rules, the evidence theory can carry out reasoning, data fusion or decision making [37]. By using information fusion technology, the learning processes of all controllers can be diagnosed in real-time by the real-time feedback gains obtained from the controllers. On this basis, the stopping criteria were designed for overlearning diagnosis of the robust MFA-IL algorithm.

The finite element (FE) method is a widely accepted and powerful tool to deal with piezoelectric smart structures. Some kinds of efficient and accurate electromechanically coupled dynamic FEs of smart structures have already been developed [38,39,40]. Among commercial FE analysis codes, ANSYS has the ability to model smart structures with piezoelectric materials, and H Karagülle et al. [41] successfully integrated vibration control actions into ANSYS modeling, where the solution was achieved as well. In this paper, ANSYS parametric design language (APDL) is used to integrate the control law into the ANSYS FE model to perform closed-loop simulations.

In this paper, using the complementary features of P-type IL control, MFA control and SM control, a robust MFA-IL control strategy was developed for the suppression of vibrations in smart structures. Due to its ability to cope with uncertainties in the learning control process, MFA control was applied to adaptively adjust the learning gain of the P-type IL control. By inserting the SM control term into the MFA control, the learning gain can be designed properly and the convergence rate of the tracking error and the robustness of the closed-loop system can be improved. A multi-source information fusion diagnosis method for the overlearning evaluation is presented based on the evidence theory, and the stopping criteria are also be designed. The proposed control method was numerically and experimentally investigated for a clamped plate under various external disturbances, and the results are illustrated and extensively discussed at the end of the present work.

The rest of this paper is organized as follows. In Section 2, based on the FE model of piezoelectric smart structures, the state space model of the equivalent linear system is developed for the purpose of control law design. The P-type IL control is employed for establishing the vibration control equations. Section 3 describes the dynamic transformation and linearization for the vibration control system. Section 4 describes the design of the robust MFA-IL control scheme. Theoretical basis of state diagnosis, based on evidence theory and the design of the stopping criteria, is introduced in Section 5. In Section 6, numerical examples are presented to demonstrate the validity of the proposed method. In Section 7, a complete active vibration control system is set up to conduct an experimental investigation. The conclusions and outlooks are drawn in Section 8.

## 2. FE Model and P-type IL Control

The linear electro-mechanically coupled dynamic FE equations of the piezoelectric smart structure can be written as [23]:

(1) $$\left[\begin{array}{cc} M_{uu} & 0\\ 0 & 0 \end{array}\right]\left\{\begin{array}{c} \ddot{u}\\ \ddot{\phi } \end{array}\right\}+\left[\begin{array}{cc} C_{uu} & 0\\ 0 & 0 \end{array}\right]\left\{\begin{array}{c} \dot{u}\\ \dot{\phi } \end{array}\right\}+\left[\begin{array}{cc} K_{uu} & K_{u\phi }\\ K_{\phi u} & K_{\phi \phi } \end{array}\right]\left\{\begin{array}{c} u\\ \phi \end{array}\right\}=\left\{\begin{array}{c} F_{u}\\ F_{\phi } \end{array}\right\}$$

where u and ϕ are the vectors of nodal displacements and electric potentials; $M_{uu}$, $C_{uu}$, $K_{uu}$, $K_{u\phi }$($K_{\phi u}$) and $K_{\phi \phi }$ are the structural mass matrix, the damping matrix, the mechanical stiffness matrix, the piezoelectric coupling matrix and the dielectric stiffness matrix, respectively; $F_{u}$ and $F_{\phi }$ are the vectors of mechanical force and electric load, respectively.

The damping matrix $C_{uu}$ is usually defined as a linear combination of the structural mass matrix $M_{uu}$ and the mechanical stiffness matrix $K_{uu}$, which is written as follows:

(2) $$C_{uu}=\alpha M_{uu}+\beta K_{uu}$$

where the constants α and β are the Rayleigh damping coefficients.

The piezoelectric sensor generates output electric potential as long as the structure is oscillating. The system (1) can be uncoupled into the following independent equations for the sensor output electric potential:

(3) $$\phi =K_{\phi \phi }^{-1}(F_{\phi }-K_{\phi u}u)$$

and the structural displacement

(4) $$M_{uu}\ddot{u}+C_{uu}\dot{u}+K^{*}u=F_{u}-K_{u\phi }K_{\phi \phi }^{-1}F_{\phi }$$

where $K^{*}=K_{uu}-K_{u\phi }K_{\phi \phi }^{-1}K_{\phi u}$.

Note that $F_{\phi }$ is usually zero in the sensor, thus, Equation (3) can be rewritten as $\phi =-K_{\phi \phi }^{-1}K_{\phi u}u$. Then, the rate of change of the sensor output electric potential can be written as:

(5) $$\dot{\phi }=-K_{\phi \phi }^{-1}K_{\phi u}\dot{u}$$

The system output error is defined as:

(6) $$e=y_{d}-y$$

where $y_{d}$ is the desired output signal and y is measured output signal. 

In the application of vibration suppression, we require the desired output signal to be zero. The measured output signal in this paper is the rate of change of the sensor output electric potential, namely, $y=\dot{\phi }$. As a discrete-time system, the output error at the kth moment can be given as:

(7) $$e(k)=0-\dot{\phi }(k)=K_{\phi \phi }^{-1}K_{\phi u}\dot{u}(k)$$

According to the update rule of the P-type IL control [42], the feedback gain at the kth moment can be given as:

(8) G(k)=G(k−1)+Φe(k−1)

where Φ and G(k) are the learning gain matrix and feedback gain matrix, respectively.

The feedback gain G(k) is stored in memory at the (k−1)th moment and applied for the next iteration when the system operates. The input voltage of the actuator is expressed as:

(9) $$V_{a}=-G\dot{\phi }$$

The electric load vector is defined as:

(10) $$F_{\phi }=C_{a}V_{a}$$

where $C_{a}$ is the capacitance constant of the piezoelectric actuator.

The control force can be defined as $F_{a}=-K_{u\phi }K_{\phi \phi }^{-1}F_{\phi }$ and by combining with Equations (5), (9) and (10), $F_{a}$ can be rewritten as:

(11) $$F_{a}=-K_{u\phi }K_{\phi \phi }^{-1}C_{a}GK_{\phi \phi }^{-1}K_{\phi u}\dot{u}$$

The control force generated by actuator is used to suppress structural vibration. Substituting Equations (5), (9) and (10) into Equation (4), the vibration control equation of the piezoelectric smart structure can be expressed as:

(12) $$M_{uu}\ddot{u}+(C_{uu}+K_{u\phi }K_{\phi \phi }^{-1}C_{a}GK_{\phi \phi }^{-1}K_{\phi u})\dot{u}+K^{*}u=F_{u}$$

## 3. Dynamic Transformation and Linearization of Vibration Control Equations

As a discrete-time system, the system (12) can be approximated by the following form at the kth moment:

(13) $$M_{uu}\ddot{u}(k)+[C_{uu}+K_{u\phi }K_{\phi \phi }^{-1}C_{a}G(k)K_{\phi \phi }^{-1}K_{\phi u}]\dot{u}(k)+K^{*}u(k)=F_{u}(k)$$

Again, using Equations (5) and (7), the system (13) can be rewritten as:

(14) $$\dot{y}(k)=-(K_{M}^{*}C_{uu}K_{\phi u}^{-1}K_{\phi \phi })y(k)-[K_{M}^{*}K_{C}^{*}G(k)]y(k)+K_{M}^{*}F^{*}(k)$$

where $K_{M}^{*}=K_{\phi \phi }^{-1}K_{\phi u}M_{uu}^{-1}$, $K_{C}^{*}=K_{u\phi }K_{\phi \phi }^{-1}C_{a}$, $F^{*}(k)=K^{*}u(k)-F_{u}(k)$.

Similar to Equation (8), a time-varying version for P-type IL updating rule is given as [42]:

(15) G(k)=G(k−1)+Φ(k−1)e(k−1)

where Φ(k−1) is the learning gain matrix, which is now time-varying.

Because $\dot{y}(k)=\frac{y(k+1)-y(k)}{T}$ for the sample period T, substituting Equation (15) into Equation (14), the discrete-time form of the system (14) can be given as:

(16) $$y(k+1)=-T[K_{M}^{*}C_{uu}K_{\phi u}^{-1}K_{\phi \phi }+K_{M}^{*}K_{C}^{*}G(k-1)+\;K_{M}^{*}K_{C}^{*}\Phi (k-1)e(k-1)-\frac{1}{T}]y(k)+TK_{M}^{*}F^{*}(k)$$

where Φ(k−1) and y(k) are the system input and the system output, respectively.

It can be known from Equation (16) that the partial derivatives of y(k+1), with respect to output y(k) and input Φ(k−1), are continuous and the system is generalized *Lipschitz*. For system (16), with $\left\|{\left[\Delta y(k),\Delta \Phi (k-1)\right]}^{T}\right\|\neq 0$ for each fixed k, there must exist Ψ(k), named the PPD matrix, such that Equation (16) can be transformed into the following equivalent full form dynamic linearization model:

(17) $$\Delta y(k+1)=\Psi (k){\left[\Delta y(k),\;\Delta \Phi (k-1)\right]}^{T}$$

where $\Psi (k)=[\varphi_{1}(k),\varphi_{2}(k)]$, $\varphi_{1}(k)$, $\varphi_{2}(k)\in R^{N\times N}$ and ‖Ψ(k)‖<b, b is a positive constant.

**Proof.**  From Equation (16) we have

(18) $$\begin{array}{l} \Delta y(k+1)=y(k+1)-y(k)\\ =\{-T[K_{M}^{*}C_{uu}K_{\phi u}^{-1}K_{\phi \phi }+K_{M}^{*}K_{C}^{*}G(k-1)+K_{M}^{*}K_{C}^{*}\Phi (k-1)e(k-1)-\frac{1}{T}]y(k)+TK_{M}^{*}F^{*}(k)\}\\ -\{-T[K_{M}^{*}C_{uu}K_{\phi u}^{-1}K_{\phi \phi }+K_{M}^{*}K_{C}^{*}G(k-1)+K_{M}^{*}K_{C}^{*}\Phi (k-2)e(k-1)-\frac{1}{T}]y(k-1)+TK_{M}^{*}F^{*}(k)\}\;\\ +\{-T[K_{M}^{*}C_{uu}K_{\phi u}^{-1}K_{\phi \phi }+K_{M}^{*}K_{C}^{*}G(k-1)+K_{M}^{*}K_{C}^{*}\Phi (k-2)e(k-1)-\frac{1}{T}]y(k-1)+TK_{M}^{*}F^{*}(k)\}\;\\ +\{-T[K_{M}^{*}C_{uu}K_{\phi u}^{-1}K_{\phi \phi }+K_{M}^{*}K_{C}^{*}G(k-2)+K_{M}^{*}K_{C}^{*}\Phi (k-1)e(k-2)-\frac{1}{T}]y(k-1)+TK_{M}^{*}F^{*}(k-1)\}\;\\ -\{-T[K_{M}^{*}C_{uu}K_{\phi u}^{-1}K_{\phi \phi }+K_{M}^{*}K_{C}^{*}G(k-2)+K_{M}^{*}K_{C}^{*}\Phi (k-2)e(k-2)-\frac{1}{T}]y(k-1)+TK_{M}^{*}F^{*}(k-1)\}\;\\ -\{-T[K_{M}^{*}C_{uu}K_{\phi u}^{-1}K_{\phi \phi }+K_{M}^{*}K_{C}^{*}G(k-2)+K_{M}^{*}K_{C}^{*}\Phi (k-1)e(k-2)-\frac{1}{T}]y(k-1)+TK_{M}^{*}F^{*}(k-1)\}\; \end{array}$$

$$\begin{array}{ll} \theta (k) & ≜\{-T[K_{M}^{*}C_{uu}K_{\phi u}^{-1}K_{\phi \phi }+K_{M}^{*}K_{C}^{*}G(k-1)+\;K_{M}^{*}K_{C}^{*}\Phi (k-2)e(k-1)-\frac{1}{T}]y(k-1)+TK_{M}^{*}F^{*}(k)\}\\ & -\{-T[K_{M}^{*}C_{uu}K_{\phi u}^{-1}K_{\phi \phi }+K_{M}^{*}K_{C}^{*}G(k-2)+\;K_{M}^{*}K_{C}^{*}\Phi (k-1)e(k-2)-\frac{1}{T}]y(k-1)+TK_{M}^{*}F^{*}(k-1)\}\; \end{array}$$

By virtue of the Cauchy differential mean value theorem, Equation (18) can be rewritten as

(19) $$\Delta y(k+1)=\frac{\partial y(k+1)}{\partial y(k)}\Delta y(k)+\frac{\partial y(k)}{\partial \Phi (k-2)}\Delta \Phi (k-1)+\theta (k)$$

where $\frac{\partial y(k+1)}{\partial y(k)}$ is the partial derivative value of y(k+1), with respect to output y(k), and $\frac{\partial y(k)}{\partial \Phi (k-2)}$ represents the partial derivative value of y(k), with respect to input Φ(k−2).

For each fixed k, we consider the following equation with the numerical matrix $H(k)\in R^{N\times N}$.

(20) $$\theta (k)=H(k){\left[\Delta y(k),\Delta \Phi (k-1)\right]}^{T}$$

Since condition $\left\|{\left[\Delta y(k),\Delta \Phi (k-1)\right]}^{T}\right\|\neq 0$, Equation (20) must have at least one solution $H^{*}(k)$. In fact, it must have an infinite number of solutions for each k.

Let

(21) $$\Psi (k)=H^{*}(k)+[\frac{\partial y(k+1)}{\partial y(k)},\frac{\partial y(k)}{\partial \Phi (k-2)}],$$

Then, we have Equation (17).

Based on Equation (17), the system (16) can be rewritten in the following dynamic linearization form:

(22) $$y(k+1)=\varphi_{1}(k)\Delta y(k)+\varphi_{2}(k)\Delta \Phi (k-1)+y(k)$$

where the values of $\varphi_{1}(k)$ and $\varphi_{2}(k)$ are dynamic changed. □

## 4. Controller Design

### 4.1. MFA Control

Consider the following control input criterion function:

(23) $$J(\Phi (k-1))={\left\|y_{d}(k+1)-y(k+1)\right\|}^{2}+\gamma {\left\|\Phi (k-1)-\Phi (k-2)\right\|}^{2}$$

where $y_{d}(k+1)$ is the desired output signal and γ>0 is a weighting constant. Φ(k−1) is the MFA control rate.

Substituting Equation (22) into Equation (23), then differentiating Equation (23) with respect to Φ(k−1), and letting it be equal zero, gives the following:

(24) $$\Delta \Phi (k-1)={\left(\varphi_{2}{\left(k\right)}^{T}\varphi_{2}(k)+\gamma I\right)}^{-1}\varphi_{2}{\left(k\right)}^{T}\times \left[y_{d}(k+1)-y(k)-\varphi_{1}(k)\Delta y(k)\right]$$

Equation (24) includes the calculation of the inverse matrix, which may cause computational burden once the I/O matrixes of the system are of a high dimension. The simplified form of Equation (24) can be expressed as follows:

(25) $$\Phi_{\mathrm{MFA}}(k-1)=\Phi_{\mathrm{MFA}}(k-2)+\frac{\rho \varphi_{2}{\left(k\right)}^{T}[y_{d}(k+1)-y(k)-\varphi_{1}(k)\Delta y(k)]}{{\left\|\varphi_{2}(k)\right\|}^{2}+\gamma }$$

where ρ∈(0,1] is a step-size constant, which is added to make Equation (25) general. 

In this paper, a modified projection algorithm is used to estimate the unknown PPD matrix:

(26) $$J(\Psi (k))={\left\|\Delta y(k)-\Psi (k){\left[\Delta y(k-1),\;\Delta \Phi (k-2)\right]}^{T}\right\|}^{2}+\mu {\left\|\Psi (k)-\hat{\Psi }(k-1)\right\|}^{2}$$

where $\Psi (k)=\hat{\Psi }(k-1)+\Delta \Psi (k)=[{\hat{\varphi }}_{1}(k-1),{\hat{\varphi }}_{2}(k-1)]+[\Delta \varphi_{1}(k),\Delta \varphi_{2}(k)]$, μ>0 is a weighting constant and $\hat{\Psi }(k-1)$ is an estimated value of Ψ(k−1).

Here, we differentiate Equation (26) with respect to Ψ(k), and letting it be equal to zero. According to the simplified form in Equation (25), we can obtain the parameters of the estimation algorithm as follows:

(27) $$\begin{array}{l} {\hat{\varphi }}_{1}(k)={\hat{\varphi }}_{1}(k-1)+\frac{\eta [\Delta y(k)-({\hat{\varphi }}_{1}(k-1),{\hat{\varphi }}_{2}(k-1)){\left(\Delta y(k-1),\Delta \Phi (k-2)\right)}^{T}]\Delta y{\left(k-1\right)}^{T}}{\mu +{\left\|\Delta y(k-1)\right\|}^{2}+{\left\|\Delta \Phi (k-2)\right\|}^{2}}\\ {\hat{\varphi }}_{2}(k)={\hat{\varphi }}_{2}(k-1)+\frac{\eta [\Delta y(k)-({\hat{\varphi }}_{1}(k-1),{\hat{\varphi }}_{2}(k-1)){\left(\Delta y(k-1),\Delta \Phi (k-2)\right)}^{T}]\Delta \Phi {\left(k-2\right)}^{T}}{\mu +{\left\|\Delta y(k-1)\right\|}^{2}+{\left\|\Delta \Phi (k-2)\right\|}^{2}} \end{array}$$

where η∈(0,1] is a step-size constant, which is added to make Equation (27) general.

### 4.2. Robust MFA-IL Control

The discrete-time SM is used to compensate the external disturbances and guarantee the fast convergence of feedback gain, which can increase the system robustness and control performance. By assuming the discrete sliding surface is

(28) s(k)=e(k)

and combining Equations (6) and (22), the above equation can be rewritten as follows:

(29) $$s(k+1)=e(k+1)=y_{d}(k+1)-\varphi_{1}(k)\Delta y(k)-\varphi_{2}(k)\Delta \Phi (k-1)-y(k)$$

The sliding reaching law is defined as follows [43]:

(30) $$\{\begin{array}{l} s(k+1)=(1-qT)s(k)-\varepsilon T\mathrm{fal}(s(k),\sigma ,\delta )\\ \delta >{\left(\frac{\varepsilon T}{1-qT}\right)}^{\frac{1}{1-\sigma }},\;0<\frac{\varepsilon T}{1-qT}<1 \end{array}$$

where $\mathrm{fal}(s(k),\alpha ,\delta )=\{\begin{array}{ll} {\left|s(k)\right|}^{\sigma }sign(s(k)), & \left|s(k)\right|\geq \delta \\ \frac{s(k)}{\delta^{1-\sigma }}, & \left|s(k)\right|<\delta \end{array}$, 0<σ<1, 0<δ<1, ε>0, q>0, 1−qT>0, T is the sample period. 

Substituting Equation (30) into Equation (29), gives the following:

(31) $$\Delta \Phi (k-1)=\varphi_{2}{\left(k\right)}^{-1}[y_{d}(k+1)-\varphi_{1}(k)\Delta y(k)-y(k)-(1-qT)s(k)+\varepsilon T\mathrm{fal}(s(k),\sigma ,\delta )]$$

Because the sliding mode reaching law is based on the transformed dynamic linearization, let $\Phi_{\mathrm{SM}}(k-1)=\Delta \Phi (k-1)$. Then, the final learning gain of the IL controller is equal to Equation (25) plus Equation (31), written as follows:

(32) $$\Phi (k-1)=\Phi_{\mathrm{MFA}}(k-1)+\Gamma \Phi_{\mathrm{SM}}(k-1)$$

where Γ is a weighting factor which is added to make Equation (32) general. $\Phi_{\mathrm{SM}}(k-1)$ is used to compensate for the input disturbance and increase the convergence rate.

**Figure 1:**
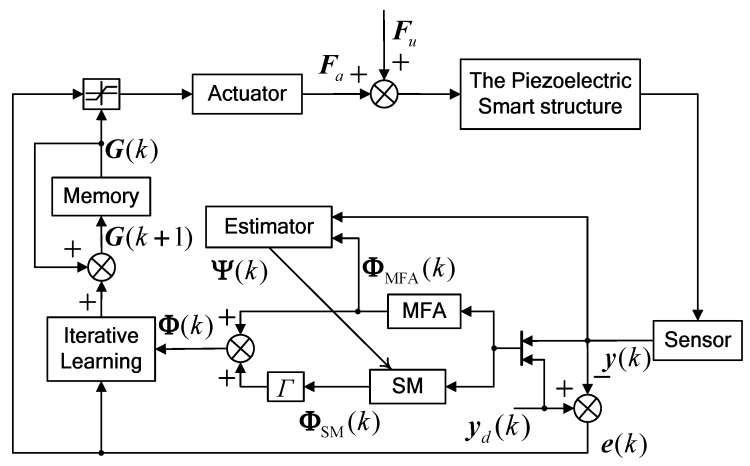
Block diagram of the robust model-free adaptive-iterative learning (MFA-IL) control.

For convenience, a block diagram of the robust MFA-IL control approach is presented in Figure 1.

## 5. The Design of the Stopping Criteria Based on Evidence Theory

### 5.1. Evidence Theory

The evidence theory is a mathematical theory and general framework for reasoning with uncertainty information in systems, which allows one to combine multiple variables from multiple sources, arriving at a degree of belief. The major definitions and concepts of the theory are briefly introduced as follows [44,45].

**Definition** **1.** *Let a finite set of elements $\Theta =\left\{Z_{1},Z_{2},\cdots ,Z_{L}\right\}$ be defined as the frame of discernment. An element can be a hypothesis, object or state. The $2^{\Theta }$, named the power set, is the set of all subsets of Θ. It is composed of each element and multi-subset and can be indicated as $2^{\Theta }=\left\{\emptyset ,\left\{Z_{1}\right\},\left\{Z_{2}\right\},\cdots \left\{Z_{1},Z_{2}\right\},\cdots ,\Theta \right\}$, where ∅ is an empty set.*

**Definition** **2.** *A mass function is a mapping of m from $2^{\Theta }$ to [0,1], and be formally defined as:*

(33) $$m:2^{\Theta }\to \left[0,1\right]$$

*Additionally, the function satisfies the following equation:*

(34) $$m(\emptyset )=0,\;\sum_{A\subseteq 2^{\Theta }}m(A)=1$$

*The function m is named basic probability assignment (BPA). m(A) expresses the proportion of all relevant and available evidence. It is claimed that a particular element of Θ belongs to the set A, but to no particular subset of A. If m(A)>0, A is called a focal element of Θ. If m(A)=0, it means that the proposition totally lacks belief.*

**Definition** **3.** *Suppose that two BPAs denoted by $m_{1}$ and $m_{2}$ are obtained from two different information sources in the same frame of discernment Θ. The degree of conflict among the evidence is denoted as follows:*

(35) $$K=\sum_{A\cap B=\emptyset }m_{1}(A)m_{2}(B)$$

*where if K=0, it means that the two pieces of evidence are fully compatible with each other. On the contrary, if K=1, it means that the two pieces of evidence totally conflict with each other. *

Dempster’s rule of combination is the most basic and widely used rule for the combination of evidence:

(36) $$\{\begin{array}{cc} \frac{1}{1-K}\sum_{A\cap B=C}m_{1}(A)m_{2}(B) & \forall C\subseteq 2^{\Theta },C\neq \emptyset \\ 0 & C=\emptyset \end{array}$$

Dempster’s combination rule is commutative and associates. Thus, the fusion result has nothing to do with the order of the fusion process. 

It has two characteristics [46]:

- Pieces of mutually supporting evidence are reinforced.
- Pieces of conflicting evidence weaken each other.

### 5.2. The Design of Stopping Criterions

Research on stopping criteria based on evidence theory in this paper involves extracting real-time feedback gains from each controller in the vibration control system, constructing the frame of discernment, choosing appropriate feature vectors that describe the learning process of the robust MFA-IL algorithm, calculating the BPAs based on the input signals of actuator, forming the fused BPAs using combination rule and diagnosing the learning states of the control method based on the BPA results.

The real-time feedback gains from each controller can be considered as a piece of evidence for diagnosing the system state, assuming M actuators are glued on the plate and that the feedback gains of the corresponding controllers are measured vectors. For the sake of simplicity, suppose that all types of states are independent of each another. Only one state can occur at any given time. Let $S_{\omega }$ represent the measurement obtained from the ωth controller (information source):

(37) $$S_{\omega }=[s_{\omega 1}\;s_{\omega 2}\;\cdots s_{\omega m_{\omega }}]\;\;\;\;\;\omega =1,2,\ldots ,M$$

where $s_{\omega i}$ is the ith element of $S_{\omega }$, $i=1,2,\ldots ,m_{\omega }$, $m_{\omega }$ is the number of elements provided by the ωth controller and $\sum_{\omega =1}^{M}m_{\omega }=n$, n is the number of features.

There are N types of states. The system states matrix can be described as [47]:

(38) $$H=\left[\begin{array}{c} X_{1}\\ X_{2}\\ \vdots \\ X_{N} \end{array}\right]=\left[\begin{array}{cccc} x_{11} & x_{12} & \cdots & x_{1n}\\ x_{21} & x_{22} & \cdots & x_{2n}\\ \vdots & \vdots & \vdots & \vdots \\ x_{N1} & x_{N2} & \cdots & x_{Nn} \end{array}\right]$$

where $X_{j}$ is a feature vector describing the jth state, $x_{ji}$ is the ith feature of the jth state, and i=1,2,…,n, j=1,2,…,N.

This paper uses the Minkowski distance for quantifying the objective evaluation of the BPAs. The Minkowski distance between $S_{\omega }$ and $X_{j}$ is reconstructed as:

(39) $$d_{\omega j}=\{\begin{array}{ll} {\left[\sum_{i=1}^{m_{\omega }}{\left(\frac{s_{\omega i}-x_{ji}}{x_{ji}}\right)}^{r}\right]}^{1/r} & \omega =1\\ {\left[\sum_{i=1}^{m_{\omega }}{\left(\frac{s_{\omega i}-x_{j\left(i+\sum_{l=1}^{\omega -1}m_{l}\right)}}{x_{j\left(i+\sum_{l=1}^{\omega -1}m_{l}\right)}}\right)}^{r}\right]}^{1/r} & \omega =2,3\ldots ,M \end{array}$$

where $d_{\omega j}$ is the distance between $S_{\omega }$ and $X_{j}$. r is a constant, such that if r=2, then the distance converges to the Euclidean distance. On the other hand, if r=1, the distance converges to the corner distance. The distances between all measurement vectors $S_{\omega }$ and all state vectors $X_{j}$ can be obtained in a matrix form:

(40) $$D=\left[\begin{array}{cccc} d_{11} & d_{12} & \cdots & d_{1N}\\ d_{21} & d_{22} & \cdots & d_{2N}\\ \vdots & \vdots & \vdots & \vdots \\ d_{M1} & d_{M2} & \cdots & d_{MN} \end{array}\right]$$

The smaller the distance $d_{\omega j}$, the more probable the jth state, based on the feedback gain acquired from the ωth controller. By defining $p_{\omega j}=1/d_{\omega j}$, a matrix form after normalizing can be expressed as follows:

(41) $$P=\left[\begin{array}{cccc} p_{11} & p_{12} & \cdots & p_{1N}\\ p_{21} & p_{22} & \cdots & p_{2N}\\ \vdots & \vdots & \vdots & \vdots \\ p_{M1} & p_{M2} & \cdots & p_{MN} \end{array}\right]=\left[\begin{array}{c} P_{1}\\ P_{2}\\ \vdots \\ P_{M} \end{array}\right]$$

where $P_{\omega }=\left[\begin{array}{cccc} p_{\omega 1} & p_{\omega 2} & \cdots & p_{\omega N} \end{array}\right]$. $P_{\omega }$ is the BPA assigned by the ωth controller to the set of states, satisfying $\sum_{j=1}^{N}p_{\omega j}=1$. 

According to Equation (35), the degree of conflicting evidence among the various controllers can be calculated. Then, the fused BPAs are computed using the combination rule, denoted in Equation (36). The fused BPAs are the pieces of evidence for state diagnosis. The threshold value was predefined as a stopping criterion. Based on the values of BPAs for a certain state, decision making on a system state can be fulfilled by comparing with the threshold value.

## 6. Numerical Examples

### 6.1. FE Model Validation

The purpose of this subsection is to examine the accuracy of the dynamic FE model established by ANSYS, by comparison of the numerical and analytical results in the open literature. 

**Figure 2:**
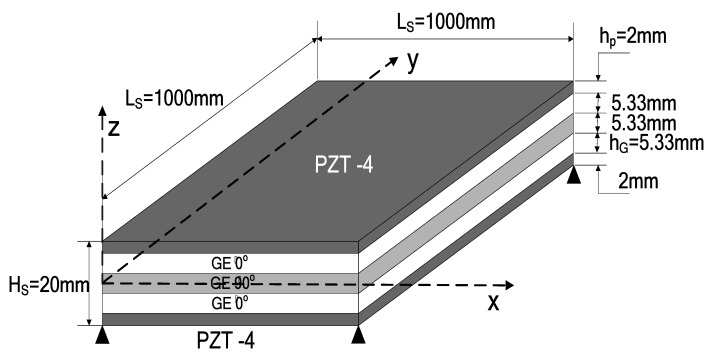
Square laminated plate.

Considering the square laminate plate, which composes of three layers of graphite-epoxy (GE, carbon-fibre reinforced) composite material (0/90/0) covered by PZT-4 piezoelectric layers poled in the z-direction (through-thickness). Then, a five-layer laminate plate (PZT-4/GE 0°/GE 90°/GE 0°/PZT-4) was formed. The geometrical data of the square laminated plate are given in Figure 2. The material properties of the GE composite material and the piezoelectric material are given in Table 1, and the densities of all materials considered are to be considered unitary ($\rho_{S}=1\;\mathrm{kg}/m^{3}$) for the purpose of comparison. The assumed boundary conditions are simply supported. The SOLID46 3-D solid element, which can be used to model the laminated composite beam or plate type structures with various layer orientations, is used for simulating the GE composite plate and the SOLID5 3-D solid element (which has a 3D piezoelectric and structural capability between the fields and is applied for simulating piezoelectric layers). 

The first five natural frequencies $\varpi_{x,y}$ can be non-dimensionalized by the expression $\lambda_{x,y}=\varpi_{x,y}L_{S}{}^{2}/H_{S}\sqrt{\rho_{S}}\times 10^{3}$. The dynamic FE numerical analysis results in this paper are compared to different approaches: (a) The finite element solution FPS (an equivalent single-layer approach and a layerwise representation of the electric potential, using the finite element method with four nodes and five degrees of freedom) by W. Larbi et al. [48]; (b) the finite element solution Q9-HSDT (higher shear deformation theory, using the finite element method with nine nodes and eleven degree of freedom) by Victor M. Franco Correia et al. [50]; (c) the finite element solutions TDST (third order shear deformation theory, using the finite element method with four nodes and seven degrees of freedom) by Tatiane Corrêa de Godoy et al. [49]; and (d) two-dimensional analytical solutions (layerwise first-order shear deformation theory and quadratic electric potential) by Ayech Benjeddou et al. [51]. The present results and their percent errors are relative to the published results (Δ%) and are shown in Table 2. It was found that a reasonably good approximation to FPS and Q9-HSDT theories was obtained, although the relative errors for some natural frequencies were greater than 5%.

### 6.2. Modeling and Choice of Controller Parameters

**Figure 3:**
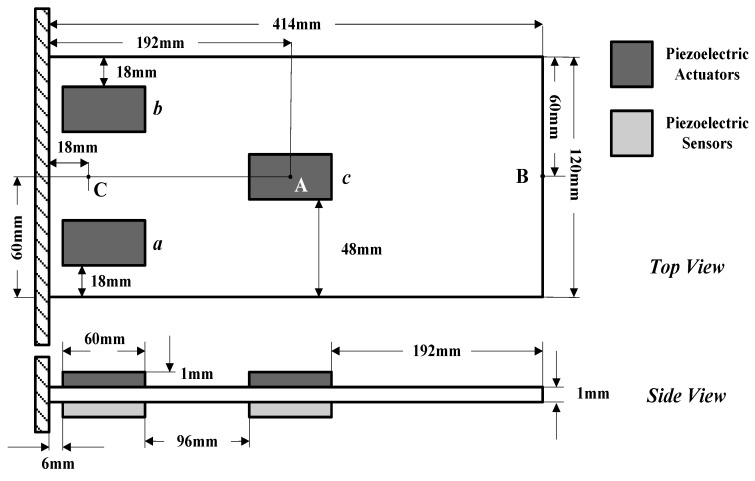
The piezoelectric smart plate.

In this section, a piezoelectric smart plate is considered for vibration control simulations. The piezoelectric smart plate is made of a host composite plate and six piezoelectric patches bonded in pairs on both sides of the plate. From Figure 3, there are three piezoelectric actuator-sensor pairs marked with *a*, *b* and *c*, respectively. The upper piezoelectric patches work as actuators to control structural vibration, while the lower ones work as sensors to obtain vibration information. The dimensions of the composite plate and the piezoelectric patches are 414 mm×120 mm×1 mm and 60 mm×24 mm×1 mm, and the configuration of the structure is shown in Figure 3. The localization of the actuator-sensor pairs is selected by referencing to [52]. The host composite plate is made of a GE composite material with five substrate layers, the stacking sequence of which is symmetric angle-ply [0/90/0/90/0]. The properties of the GE composite material and the piezoelectric material are listed in Table 3. The locations of points A, B and C are shown in Figure 3. A clamped boundary condition is assigned in the root of the piezoelectric smart plate. 

In this study, the layered solid element SOLID46 was used to model the composite plate, while SOLID5 was used to model the piezoelectric patches. The composite plate was meshed with 69 × 20 × 1 elements, and each piezoelectric patch was meshed with 10 × 4 × 1 elements. It was assumed that the same magnitude but the opposite electric field direction was applied to the upper and lower piezoelectric patches. The degrees of electric freedom for the nodes at the top and bottom surfaces of the piezoelectric patches were coupled by using the ANSYS command CP. Model analysis was implemented to find out the natural frequencies of the plate and determine the sampling period for the closed-loop system simulations [28]. The numerical and experimental results of the first three natural frequencies are given in Table 4. The sampling period was defined as $T=1/(20f_{1})$, where $f_{1}$ is the first natural frequency. The Rayleigh damping coefficients were considered as α=0.003 and β=0.0015.

The initial value of feedback gain G(k) in Equation (15) was assumed to be zero. The parameters of the robust MFA-IL controller are given in Table 5. There are three actuator-sensor pairs in the system, and the corresponding controllers offer measurements. The frame of discernment for system state diagnosis was constructed with two types of states, namely the normal learning state and the learning stopping state. The feedback gains of the controllers were chosen as the feature parameters. Let r=2 in Equation (39) and the distance converges to the Euclidean distance. The BPAs can be calculated using the method based on information sources in Section 5.2. The stopping criteria of the robust MFA-IL control algorithm are defined as follows: (a) According to Dempster’s rule of combination, each row vector of the BPA matrix P can be fused. The threshold value was set 0.985. If the fused BPA is higher than the threshold value, the learning processes of all controllers should be stopped. (b) Controllers connected to the actuators at different locations may lead to distinct learning speeds. To make all controllers learn sufficiently, the threshold value for the BPAs of each controller should also be proposed. If the BPAs from a certain controller are higher than 0.800, the learning process of the corresponding controller should be stopped. If one of the above conditions is met, the learning process of the controller is then terminated. Otherwise, the controller is in normal learning state. Considering the vibrations generated by various external disturbances, different simulations were investigated to evaluate the effectiveness of the proposed method. In the design of the P-type IL controllers, the maximum number of iterations was limited to 500 as the stopping criterion, and the fixed learning gains were selected as $\Phi_{1}=0.063$ and $\Phi_{2}=0.100$ for various cases.

### 6.3. Harmonic Excitation

In this case, the first mode control was tested by applying the harmonic force $f(t)=6\cos(\omega_{1}t)N$ at the point C, where $\omega_{1}=17.083\;\mathrm{rad}/\text{s}$ (5.4377 Hz). Due to the symmetry of the piezoelectric smart structure and the excitation location, sensors *a* and *b* have the same control feedback signals in the process of vibration.

**Figure 4:**
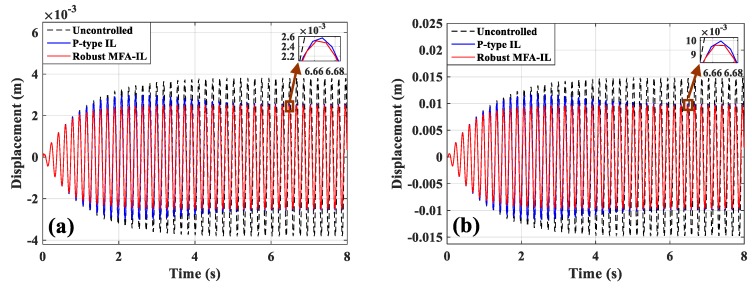
Displacement responses: (**a**) Point A, (**b**) point B.

The displacement responses of points A and B are displayed in Figure 4a,b, where it can be seen that both the robust MFA-IL control and the P-type IL control can effectively suppress the first mode vibration. The control effectiveness of the actuators is obvious at the positions with sensors (e.g., point A) and without sensors (e.g., point B). However, it is noteworthy that these results are different from Saleh′s [13], which claimed that the P-type IL control was able to compensate for the unwanted vibration at the observation point, while not being effective at other points. In addition, it was also pointed out that the P-type IL method cannot effectively attenuate the amplitude as long as the smart structure is excited by its first natural frequency. 

An effective control system has the ability to suppress the structural vibration of the whole plate rather than a small portion of the plate area. The control strategy plays an important role in designing a vibration control system for obtaining a desired performance. Besides, the locations and sizes of the piezoelectric actuators and sensors should also be seriously considered [53]. The areas of the structure where the mechanical strain is highest are always the best locations for actuators and sensors. To guarantee the actuators generate the desired control forces to suppress structural vibration, the dimensions of the actuators should also be designed appropriately. The sizes of sensors should also be chosen properly, so that accurate information of structural deformation can be obtained. A misread or incorrect sensor measurement signal may lead to unreasonable measurements and inappropriate control force generation, which will deteriorate the dynamic behavior. As long as the locations and the sizes of the actuators and sensors are selected appropriately, the P-type IL control presents good performance on first mode control. Furthermore, both the locations with sensors and the positions without sensors on the smart plate can provide good controllability of structural vibration.

**Figure 5:**
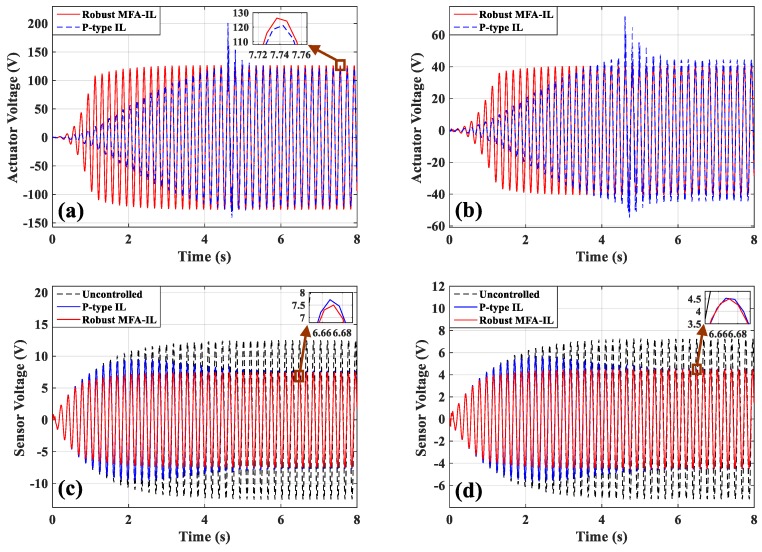
The time-domain responses of actuators/sensors: (**a**) Actuator *a*/*b*, (**b**) actuator *c*, (**c**) sensor *a*/*b*, (**d**) sensor *c*.

The displacement responses of points A and B are given in Figure 4a,b. The output voltages of sensor *a*/*b* and sensor *c* are shown in Figure 5c,d. By comparing with the P-type IL approach, a smaller amplitude can be obtained when the system is controlled by the robust MFA-IL method. In this paper, the root mean square (RMS) values of the amplitude at points A and B and output electric potential are used to quantitatively evaluate the control performance of the robust MFA-IL control and the P-type IL control. The data used to calculate the RMSs were recorded after all the controllers stopped learning, and the RMSs of amplitude are given in Table 6. 

**Figure 6:**
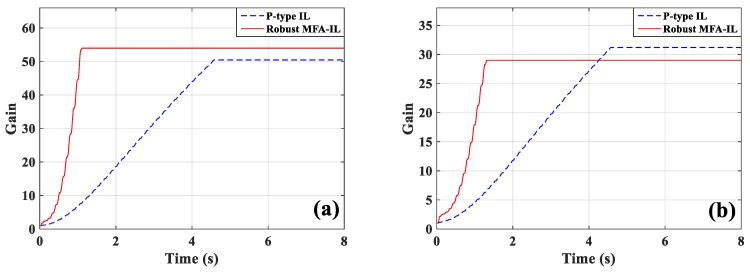
The learning processes of feedback gains: (**a**) Actuator *a*/*b*, (**b**) actuator *c*.

The control voltages applied to actuators *a*/*b* and actuator *c* are shown in Figure 5a,b. In the process of iterative learning, the actuation voltages changed sharply under the control of the P-type IL algorithm at 4.3 s. After the learning was terminated, the amplitude recovered to a smooth value. The feedback gain of the controllers at different locations had a distinct convergence rate, which may cause the control force produced by controllers to mismatch among each other. If an actuator fails to perform as expected, the performance of its neighboring actuators will be negatively affected. In order to avoid this problem, more iterations are necessary to reach satisfying level of control stability. A smaller iterative number may directly cause control spillover or even system instability. The controllers connected with actuators *a*/*b* have the same learning processes, as shown in Figure 6a. Figure 6b presents the learning processes of the feedback gains in actuator *e*. Observing the results of Figure 5 and Figure 6, the robust MFA-IL control has a faster convergence rate when compared with the P-type IL method, and the input voltages of the actuators change smoothly. Therefore, it can be seen that the proposed method enables the system to enhance control effectiveness and maintain superior stability.

**Figure 7:**
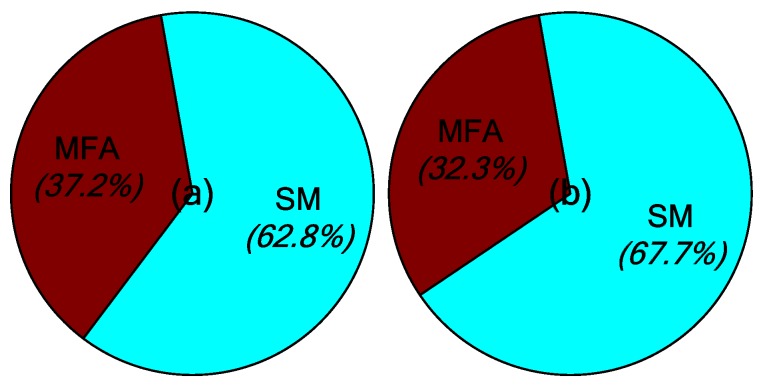
The contribution percentages of various methods to the feedback gains: (**a**) Actuators *a*/*b*, (**b**) actuator *c*.

The main contribution of the robust MFA-IL control is the improvement of learning speed. Two parts influence the learning speed of the proposed algorithm: The MFA control and the SM control. Both of these two methods accelerate the convergence speed of the feedback gains in the learning processes. Their contribution percentages to the feedback gains are shown in Figure 7, where it can be seen that the SM control plays a more important role than the MFA control.

**Figure 8:**
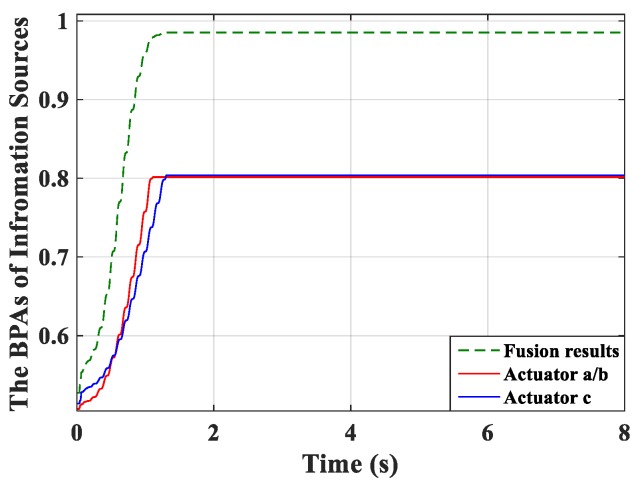
The basic probability assignment (BPA) curves.

Real-time monitoring for the fused BPA results and the BPA from each controller was implemented and is shown in Figure 8. The BPAs update at each period and the monitoring curve moves forward over time. First, the BPAs obtained from actuator *c* meet the stopping criteria. After a short period, the controller connected with actuators *a*/*b* stops learning. All controllers can learn sufficiently based on the evidence theory, and thus preferable control performance can be obtained.

### 6.4. Random Excitation

**Figure 9:**
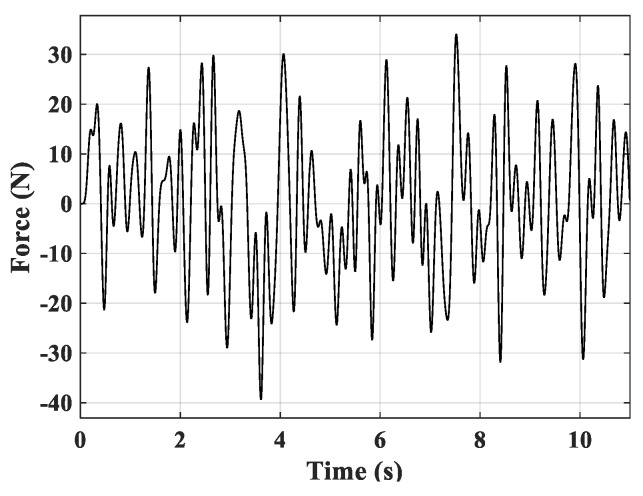
The random excitation.

In the last simulation, a random excitation (shown in Figure 9) was applied to point C to drive the piezoelectric smart plate.

**Figure 10:**
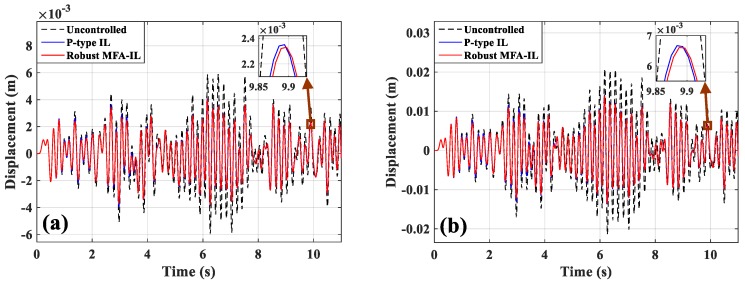
Displacement responses: (**a**) Point A, (**b**) point B.

**Figure 11:**
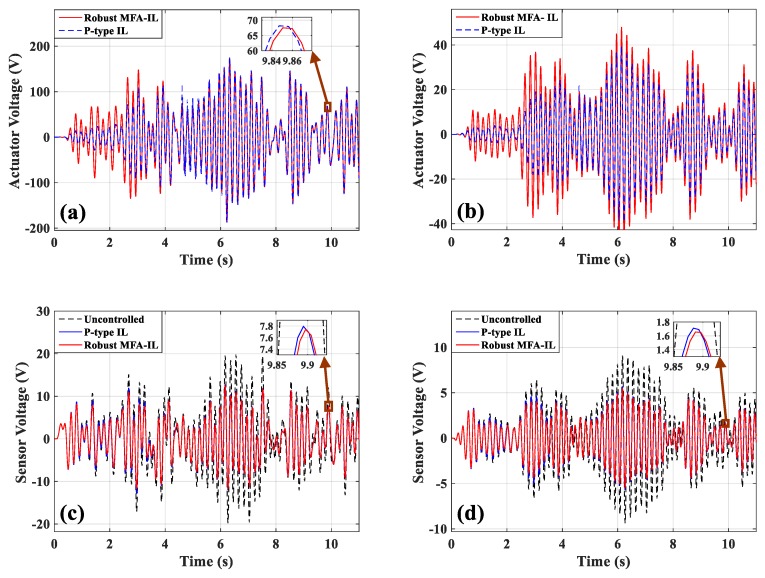
The time-domain responses of actuators/sensors: (**a**) Actuators *a*/*b*, (**b**) actuator *c*, (**c**) sensors *a*/*b*, (**d**) sensor *c*.

Figure 10a,b presents the dynamic displacement responses of points A and B, respectively. The control voltages applied on actuators *a*/*b* and actuator *c* are depicted in Figure 11a,b. The output signals of the corresponding sensors are shown in Figure 11c,d. By comparing with the robust MFA-IL method, the actuation voltage amplitudes controlled by the P-type IL method are smaller in the initial stage of simulation. Under the control of the P-type IL algorithm, the actuators cannot work effectively to consume the energy of the vibration system in the initial period. According to Figure 10 and Figure 11, it can be seen that the P-type IL control cannot suppress structural vibration in the short term. Since several hundreds of iterations lead to convergence rate of feedback gain slow. However, the learning speed of the robust MFA-IL algorithm is faster than that of the P-type IL method which has fixed gain. The time-varying learning gain is updated using I/O data, which can reflect the system dynamic behavior in real-time.

**Figure 12:**
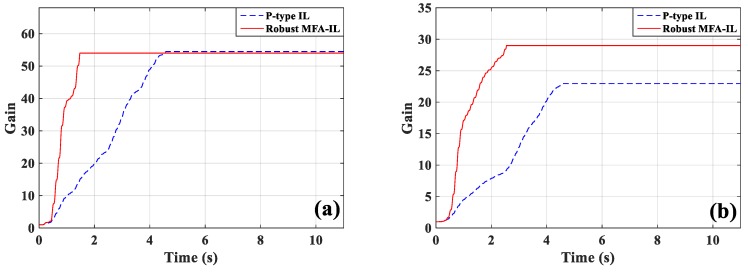
The learning processes of feedback gains: (**a**) Actuators *a*/*b*, (**b**) actuator *c*.

**Figure 13:**
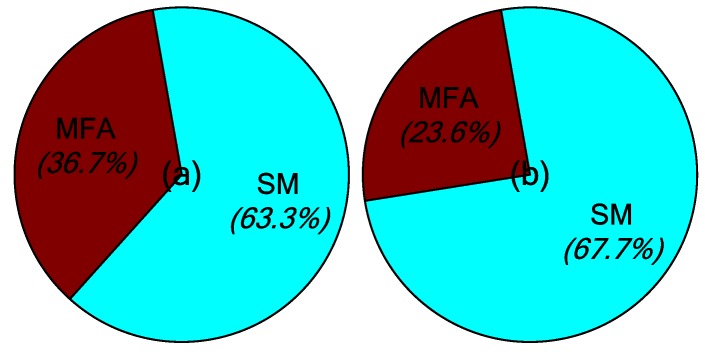
The contribution percentages of various methods to feedback gain: (**a**) Actuators *a*/*b*, (**b**) actuator *c*.

The learning processes for feedback gain for the controllers connected actuators *a*/*b* and actuator *c* are shown in Figure 12. The percentages contributed by the MFA control and the SM control are shown in Figure 13, where it can be seen that the SM control has a larger impact on the convergence rate of feedback gain than the MFA control.

**Figure 14:**
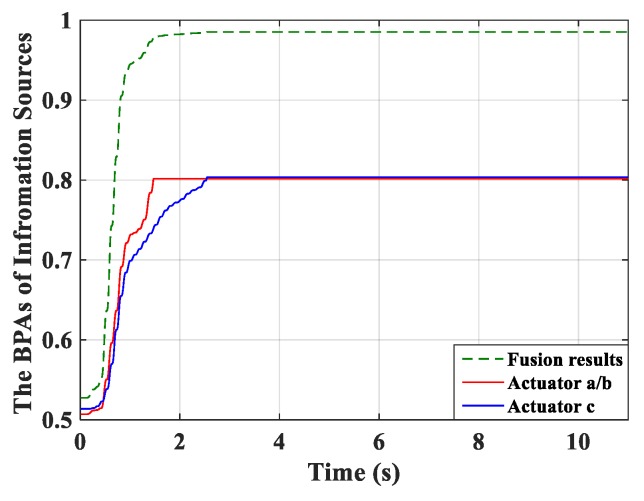
The BPAs curves.

The RMSs of amplitude were calculated and are shown in Table 6. A similar control effect to the previous simulation can be observed. The robust MFA-IL control in this simulation presents a better control performance and makes the learning speed of the controller faster by comparing with the P-type IL control. The real-time monitoring results of the fused BPAs and the BPAs from each controller are depicted in Figure 14.

**Figure 15:**
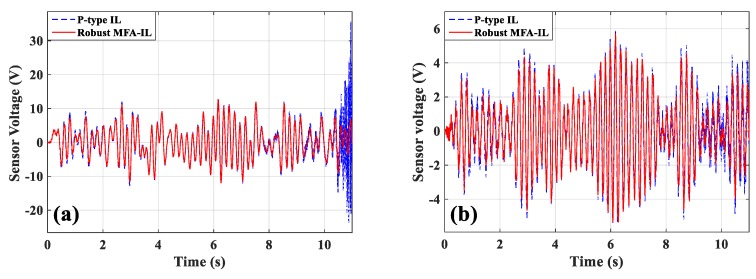
The time-domain responses of sensors: (**a**) Sensors *a*/*b*, (**b**) sensor *c*.

In order to test the robustness of the proposed method, the harmonic noise signal f(t)=cos(152⋅t)N was added to the external excitation at point C. The controller’s parameters were set up the same as mentioned above. Figure 15 shows the sensor output signals. The added noise resulted in a decrease of system performance and divergence as long as the system was controlled by the P-type IL method, while the robust MFA-IL control could maintain the stability of the control system. These comparative results validate that the proposed control method possesses excellent control performance and robustness to the noise from external excitation. 

## 7. Experimental Investigations

### 7.1. Experimental Model

**Figure 16:**
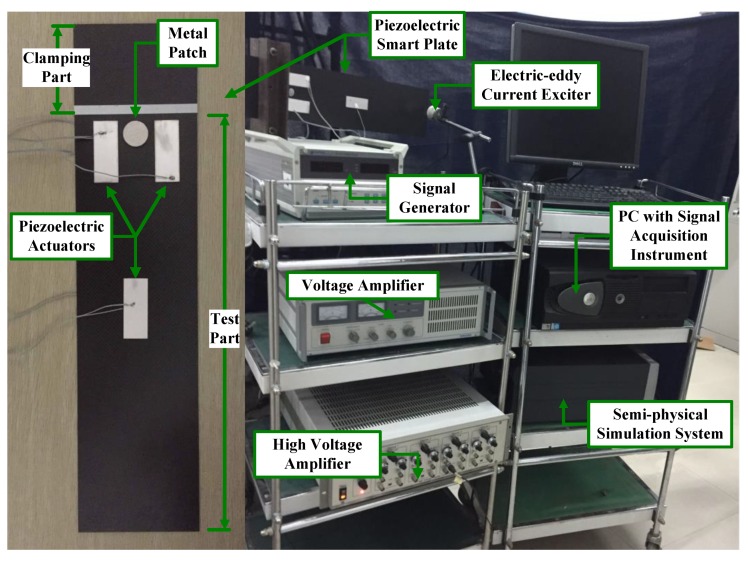
Experimental devices and the specimen.

To verify the feasibility and the performance of the robust MFA-IL algorithm, an active vibration control system was established. The experimental devices are illustrated in Figure 16. The robust MFA-IL control algorithm and the P-type IL control algorithm were implemented in a MATLAB/Simulink environment. The real-time code was automatically operated in the real-time semi-physical simulation system. The dimensions of the specimen were the same as in the numerical simulations above, and exciting position point C was replaced by a metal patch. The experimental setup was composed of a piezoelectric smart plate, an electric-eddy current exciter (JZF-1, Beijing, China), a voltage amplifier (YE5872A, State College, PA, USA), a high voltage amplifier (E70, Harbin, China), a real-time semi-physical simulation system (Quarc, Toronto, ON, Canada) and a personal computer (PC) with a signal acquisition instrument. The piezoelectric patches were glued on the host plate using commercial cyanoacrylate glue. 

**Figure 17:**
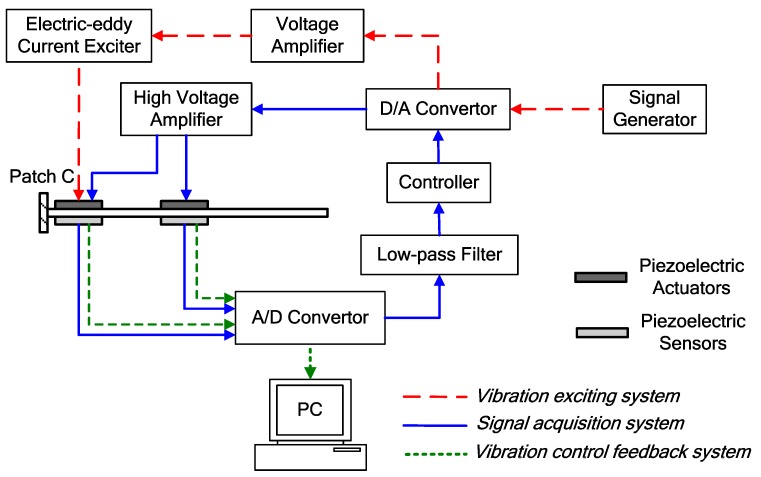
Block diagram of the experimental system.

A block diagram of the experimental system is illustrated in Figure 17. The experimental system includes three subsystems, namely: The vibration exciting system, the signal acquisition system and the vibration control feedback system. The signal transfer paths of these three subsystems are marked by red, green and blue color with different line types.

For the vibration exciting system, the excitation signal was produced by the signal generator, which was used to simulate the external excitation of the piezoelectric smart plate. After digital to analog (D/A) conversion, the excitation signal was amplified by the voltage amplifier. The signal was transmitted to the electric-eddy current exciter, which could then convert the electrical signal into the vibration excitation.

For the signal acquisition system, three piezoelectric sensors (sensors *a*, *b* and *c*) were used to obtain the vibration information of the piezoelectric smart plate. The dynamic signals from the piezoelectric sensors were sent to the PC after analog to digital (A/D) conversion, which were then used to estimate the performance of the investigated control algorithms.

For the vibration control feedback system, the dynamic signals from the piezoelectric sensors were fed to a A/D convertor. To remove high-frequency noise, the input signals were passed through a low-pass filter set to 14 Hz. The controllers conduct both signal processing and controlling design in the MATLAB/Simulink 2014a environment. Running the control algorithms, the control signals were computed and sent through the D/A convertor. After amplification from the high voltage amplifier, the control signals were applied to the actuators for suppressing structural vibration. The experimental sample period was specified as 3 ms. 

### 7.2. Modal Identification

**Figure 18:**
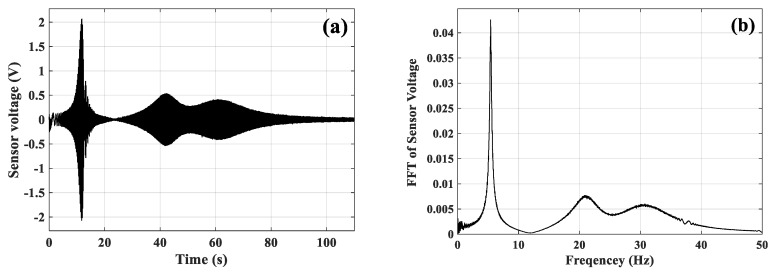
Measured vibration responses excited by actuator *a*: (**a**) Time-domain responses of sensor *a*, (**b**) frequency responses of sensor *a*.

A chirp signal is utilized to determine the natural frequencies of the piezoelectric smart plate. Actuator *a* was excited by a chirp signal with an amplitude of 100V, while the output voltages of the Sensor *a* were stored after filtering. The starting frequency was 0.5 Hz, and the stopping frequency was 50 Hz. The sweep time was 100 s. Then, the fast Fourier transform (FFT) of the time response was calculated. Figure 18b depicts the frequency responses of sensor *a* when applying FFT to the time-domain signal plotted in Figure 18a. The experimental natural frequencies obtained from the FFT plot are given in the same table (Table 4) for comparison. The biggest difference, 13.9%, occurs in the second natural frequency. 

Conditions that may lead to the difference of modal frequencies between the numerical solutions and experimental solutions are considered as follows: (1) The clamp-free boundary condition in the simulations is ideal. While in the experiments, the boundary of the plate may not be totally clamped. (2) The geometric parameters and material properties of the piezoelectric smart plate cannot be known accurately enough. The parameters used in the numerical simulations are not precisely consistent with those of the plate applied in the experimental material. (3) The mass of both the glue and the connected signal wire of the piezoelectric patches are not considered in the simulations. The modal analysis in simulations is used to provide an approximate solution for verifying the feasibility of the control algorithms, the difference of modal frequencies between the numerical results and the experimental results is acceptable. It is clear that the FE model sufficiently predicts the natural frequencies of the piezoelectric smart plate. Filters were designed and utilized in the experiments to deal with high frequency noise. For investigating the control algorithms, the designed low-pass filters were applied. The cutoff frequency of the low-pass filters was specified at 30 Hz. 

### 7.3. Experiments Results and Discussions

**Figure 19:**
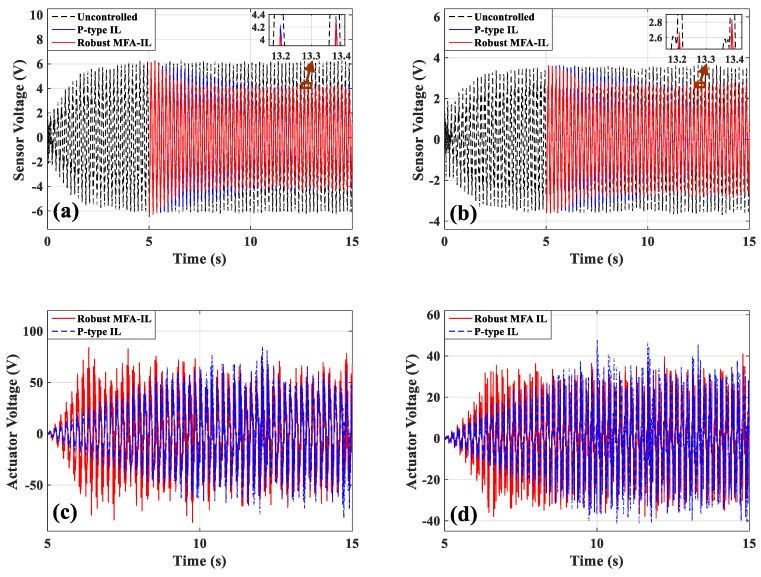
The time-domain response of actuators/sensors: (**a**) Actuators *a*/*b*, (**b**) actuator *c*, (**c**) sensors *a*/*b*, (**d**) sensor *c*.

During the experiments, the robust MFA-IL control and P-type IL control algorithms were used to suppress the structural vibration of the cantilevered plate. The first mode control was tested in this section, where the parameters of the P-type IL controller are chosen as follows: The fixed learning gain Φ=0.54. The maximum number of iterations was set to 1500 as to avoid control spillover or system instability. The parameters of the robust MFA-IL controller are listed in Table 7. The stopping criteria in the experiment are the same with those in Section 6.2. The sensor output signals (shown in Figure 19a,b) were moved forward to compensate for the phase lag effect. Five seconds after harmonic excitation driving, the proposed control algorithms were implemented to suppress the structural vibration. 

**Figure 20:**
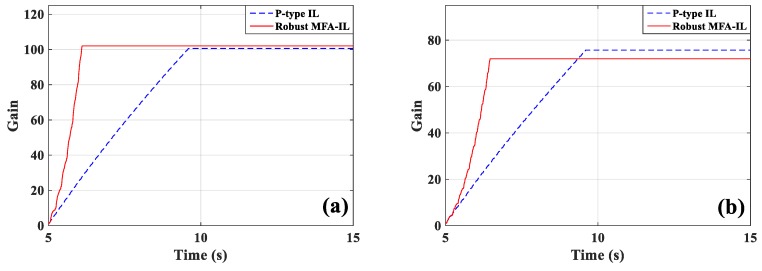
The learning processes of feedback gains: (**a**) Actuators *a*/*b*, (**b**) actuator *c*.

The measured signals of sensors *a*/*b* and sensor *c*, and the corresponding actuation voltages obtained from the robust MFA-IL controller and the P-type IL controller are shown in Figure 19. The sensor *a*/*b* and sensor *c* output signals used to calculate the RMS values were recorded after learning termination. The RMS values (shown in Table 6) were used to quantitatively evaluate the control performance of the various methods. From the RMS values, it can be seen that the P-type IL control provides a 31.9% reduction and the robust MFA-IL control provides a 33.73% reduction. It is clear that the robust MFA-IL control is more effective at reducing the vibration amplitude of the flexible plate than the P-type IL control. It can be concluded that the proposed control method exhibits excellent performance when integrating MFA control and SM control. The learning processes of the feedback gains are shown in Figure 20. Under the control of the robust MFA-IL method, the structural vibration can be suppressed faster when compared with the robust MFA-IL algorithm. 

**Figure 21:**
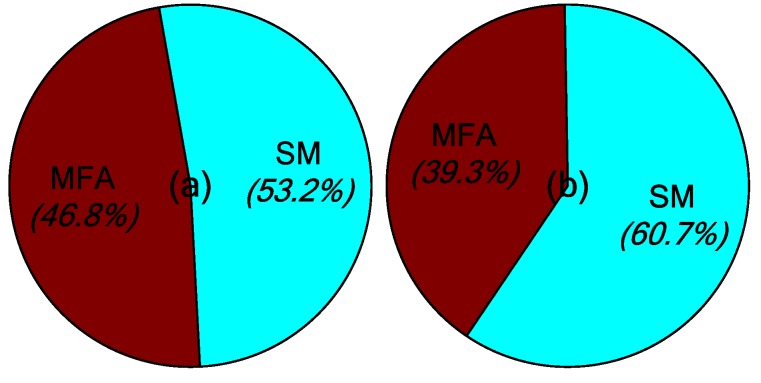
The contribution percentages of various methods to feedback gain: (**a**) Actuators *a*/*b*, (**b**) actuator *c*.

**Figure 22:**
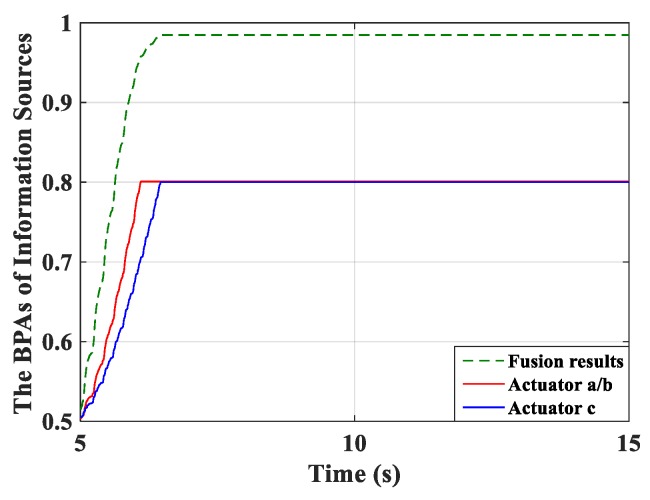
The BPAs curves.

The percentages of contributed feedback gain from the MFA and SM methods are shown in Figure 21, where it can be seen that SM control has a larger impact on the convergence speed of feedback gain than MFA control. The real-time monitoring curves of the fused BPA results and the BPAs from each information source are shown in Figure 22. By applying evidence theory to the design of the stopping criteria, the learning processes of all controllers can be evaluated in real-time. The decision-making based on the appropriate stopping criteria makes all controllers learn sufficiently, so that a better control performance is obtained.

Comparing the experimental results with the numerical results, it can be seen that the vibration suppression trends performed similarly. Due to the difference of the sample period and the unknown nature of the experimental system, the parameters selected in the simulations and experiments are different.

## 8. Conclusions and Outlooks

A robust MFA-IL control method was developed for the active vibration control of piezoelectric smart structures. Considering the uncertainty of interaction among all actuators in the control process, the MFA control was incorporated into P-type IL control for the adjustment of learning gain. In order to achieve a fast control response and enhance the stability of the system, SM control was adopted to ensure a fast dynamic response and compensate for the influence of uncertain noise. The multi-source information fusion method based on the evidence theory was adopted to design the stopping criteria of the robust MFA-IL method. The vibration control equations of piezoelectric smart structures were derived from the dynamic FE equations of a linear elastic system. The dynamic linear method was applied to transfer the vibration control equations for the design of the robust MFA-IL controller. The simulation and experimental results were presented and compared with the corresponding results using the P-type IL control approach. 

As long as the locations and sizes of actuators and sensors are chosen appropriately, both P-type IL control and robust MFA-IL control can effectively suppress structural vibration when the piezoelectric smart plate is excited by its first natural frequency. Furthermore, the whole structure presents good controllability, rather than small portions bonded with piezoelectric sensors, using both the P-type IL method and the robust MFA-IL method. 

The robust MFA-IL control method accelerates the learning speed of the controller. Based on the comprehensive comparative analysis of the numerical and experimental results, the proposed control method can achieve better control performance and is more robust to external disturbances when compared with the P-type IL control. In other words, the proposed control method overcomes the inherent drawbacks of P-type IL control and achieves the desired control performance.

Although the robust MFA-IL control method was applied for a plate structure in this paper, considering its advantages presented above, this approach can be applied for other structures, like beam structures, extending the possibilities of engineering and research applications. Dynamic linearization was an effective method in developing the proposed method for nonlinear systems. Dynamic linearization mainly introduces optimal technology as a tool for the controller design and analysis. In future work, it will be expected that the dynamic linearization method will be combined with an online model identification technique to deal with much more practical problems, those typically encountered in industrial applications. This may result in a simpler controller structure and obtain a better degree of control precision.

## Figures and Tables

**Table 1:** Properties of the graphite-epoxy (GE) composite material and the piezoelectric material.

GE [48]	PZT-4 [49]
Yong’s modulus (GPa)	Elastic stiffness (GPa)
$E_{11}=132.38$;	$C_{11}=138.50$; $C_{12}=77.37$;
$E_{22}=E_{33}=10.76$;	$C_{13}=73.64$; $C_{33}=114.75$;
Shear modulus (GPa)	$C_{44}=25.60$; $C_{66}=30.60$;
$G_{12}=G_{13}=5.65$;	Piezoelectric stain ($C/m^{2}$)
$G_{23}=3.61$;	$e_{16}=e_{25}=12.72$;
Poisson’s ratio	$e_{31}=e_{32}=-5.2$;
$v_{12}=v_{13}=0.24$;	$e_{33}=15.08$;
$v_{23}=0.49$;	Permittivity (F/m)
-	$\varepsilon_{11}=\varepsilon_{22}=1.305\times 10^{-8}$;
-	$\varepsilon_{33}=1.151\times 10^{-8}$;

**Table 2:** First five frequencies parameters.

Mode	Present	FPS [48]	Q9-HSDT [50]	TSDT [49]	2D [51]
1	232.62	231.42 (0.52%)	230.46 (0.94%)	225.98 (2.94%)	246.07 (−5.47%)
2	523.90	521.88 (0.39%)	520.38 (0.68%)	542.29 (−3.39%)	559.62 (−6.38%)
3	665.75	667.91 (−0.32%)	662.91 (0.43%)	680.11 (−2.11%)	693.60 (−4.02%)
4	916.35	909.34(0.77%)	908.46 (0.87%)	906.09 (1.13%)	967.14 (−5.25%)
5	1023.40	1027.22 (−0.37%)	1022.09 (0.13%)	1099.05 (−6.88%)	1091.46 (−6.19%)

**Table 3:** Properties of the GE composite material and the piezoelectric material.

GE	PZT-5H
Yong’s modulus (GPa)	Elastic stiffness (GPa)
$E_{11}=40.51$;	$C_{11}=126$; $C_{12}=79.5$;
$E_{22}=E_{33}=13.96$;	$C_{13}=84.1$; $C_{33}=117$;
Shear modulus (GPa)	$C_{44}=23.3$; $C_{66}=23$;
$G_{12}=G_{13}=3.1$;	Piezoelectric stain ($C/m^{2}$)
$G_{23}=1.55$;	$e_{16}=e_{25}=17$;
Poisson’s ratio	$e_{31}=e_{32}=-6.5$;
$v_{12}=v_{13}=0.22$;	$e_{33}=23.3$;
$v_{23}=0.11$;	Permittivity (F/m)
Density ($\mathrm{kg}/m^{3}$)	$\varepsilon_{11}=\varepsilon_{22}=1.503\times 10^{-8}$;
ρ=1830	$\varepsilon_{33}=1.3\times 10^{-8}$;
-	Density ($\mathrm{kg}/m^{3}$)
-	ρ=7500

**Table 4:** First three natural frequencies of the piezoelectric smart plate.

Mode	Numerical (Hz)	Experimental (Hz)	Error
1	5.438	5.326	2.1%
2	24.217	21.259	13.9%
3	28.683	31.593	−9.2%

**Table 5:** The control parameters.

Parameter	Value	Parameter	Value
λ	1	α	0.5
ρ	0.1	δ	1.5
μ	1	ε	6
η	0.1	q	60
-	-	Γ	0.5

**Table 6:** Root mean square (RMS) values of amplitude.

Algorithm	Case 1				Case 2				Experiment	
	Point A	Point B	Sensor *a*/*b*	Sensor *c*	Point A	Point B	Sensor *a*/*b*	Senso *c*	Sensor *a*/*b*	Sensor *c*
Uncontrolled	0.0027	0.0106	8.9034	5.1768	0.0024	0.0087	8.1071	3.9265	3.7644	2.1672
P-type IL	0.0018	0.0071	5.4976	3.2501	0.0016	0.0055	5.0016	2.3231	2.5570	1.4806
Robust MFA- IL	0.0017	0.0067	5.2075	3.0731	0.0015	0.0053	4.7678	2.2119	2.4876	1.4510

**Table 7:** The control parameters.

Parameter	Value	Parameter	Value
λ	1	α	0.5
ρ	0.1	δ	1.5
μ	1	ε	16
η	0.05	q	150
-		Γ	0.8
